# The Role of Therapeutic Plasma Exchange in the Management of Myeloma-Related Cast Nephropathy: A 10-Year Real-World Cohort Study

**DOI:** 10.3390/jcm15020417

**Published:** 2026-01-06

**Authors:** Hasan Salur, Unal Atas, Nurcan Alhan, Ece Vural, Utku Iltar, Orhan Kemal Yucel, Ozan Salim

**Affiliations:** 1 Department of Internal Medicine, Antalya City Hospital, Antalya 07080, Turkey; hasansalur2013@gmail.com; 2 Faculty of Medicine, Department of Internal Medicine, Division of Hematology, Akdeniz University, Antalya 07100, Turkey; vrlunalatas@gmail.com (U.A.); okyucel@hotmail.com (O.K.Y.); ozansalim@gmail.com (O.S.); 3 Education and Research Hospital Hematology Clinic, Alanya Alaaddin Keykubat University, Antalya 07425, Turkey; nurcanalhan@gmail.com; 4 Clinic of Hematology, Ankara Liv Hospital, Ankara 07425, Turkey; vrlece@gmail.com

**Keywords:** multiple myeloma, cast nephropathy, therapeutic plasma exchange, free light chains, renal impairment, dialysis dependence

## Abstract

**Background:** Renal impairment is a frequent and severe complication of multiple myeloma, most commonly caused by light-chain cast nephropathy. Therapeutic plasma exchange (TPE) has been proposed as an adjunctive approach to rapidly reduce circulating free light chains; however, its clinical benefit remains controversial. **Methods:** We retrospectively analyzed 71 patients treated between 2013 and 2023, of whom 30 received TPE in addition to anti-myeloma therapy and 41 received anti-myeloma therapy alone. Renal outcomes were assessed within a predefined early treatment window encompassing the first 4–6 cycles of therapy. Renal response was defined as a ≥50% reduction in serum creatinine and/or dialysis independence. Multivariable logistic regression and sensitivity analyses were performed to adjust for baseline imbalances, including renal function and anti-myeloma backbone therapy. **Results:** Although renal function improved significantly over time in both groups, renal response rates were comparable between patients treated with and without TPE (40% vs. 36.6%). In multivariable analysis, TPE was not independently associated with renal response. Importantly, in a sensitivity analyses restricted to patients receiving bortezomib-based regimens, the addition of TPE remained unassociated with improved renal outcomes. **Conclusions:** In this real-world cohort, adjunctive TPE did not confer a significant advantage in renal recovery or dialysis independence beyond contemporary anti-myeloma therapy. These findings indicate that renal recovery is predominantly driven by effective anti-myeloma treatment rather than extracorporeal light-chain removal.

## 1. Introduction

Multiple myeloma (MM) is a clonal plasma-cell malignancy characterized by the overproduction of monoclonal immunoglobulins and/or free light chains (FLCs) [1]. Renal dysfunction is a common and clinically significant manifestation of MM, occurring in nearly one-third of newly diagnosed patients [2]. The most common cause of renal dysfunction in MM is cast nephropathy, a pathologic entity resulting from intratubular precipitation of monoclonal FLCs with Tamm–Horsfall proteins, leading to tubular obstruction, inflammation, and acute kidney injury (AKI) [3,4,5]. Rapid diagnosis and early initiation of effective therapy are critical; as renal recovery is strongly associated with improved survival.

The cornerstone of treatment for light-chain cast nephropathy is rapid suppression of monoclonal light-chain production through effective anti-myeloma therapy, including proteasome inhibitors, immunomodulatory drugs, and dexamethasone. Among these, bortezomib-based regimens have become standard due to their rapid cytoreductive activity and suitability even in patients with severe renal failure. However, before anti-myeloma agents achieve adequate biochemical control, circulating FLCs may continue to cause ongoing tubular injury. This observation has prompted interest in extracorporeal strategies aimed at accelerating light-chain removal [6].

Therapeutic plasma exchange (TPE) has been proposed as a supportive intervention to accelerate clearance of circulating FLCs, thereby reducing tubular precipitation and improving renal recovery. Early single-center studies suggested potential benefit, reporting reductions in serum light-chain levels and improvements in renal function [6,7]. However, subsequent evidence has been inconsistent [8,9]. Notably, the randomized controlled trial by Clark et al. failed to demonstrate a reduction in death, dialysis dependence, or severe renal dysfunction at 6 months in patients treated with TPE in addition to conventional therapy [9]. Conversely, Leung et al. reported improved renal parameters following TPE in biopsy-proven cast nephropathy [10].

Given these conflicting data, the role of TPE in MM-associated cast nephropathy remains controversial. Recent guidelines acknowledge the theoretical rationale for extracorporeal removal of light chains but highlight the absence of robust clinical evidence supporting routine use. Moreover, real-world practice varies widely across centers, with some clinicians reserving TPE for selected patients with biopsy-confirmed cast nephropathy, high circulating light-chain burden, or rapidly progressive in renal dysfunction.

The therapeutic benefit of TPE in cast nephropathy secondary to multiple myeloma remains incompletely defined, largely due to limited and heterogeneous data. Accordingly, evaluating real-world outcomes of TPE in this setting is essential to inform clinical decision-making and optimize patient care.

We hypothesized that, in an unselected real-world cohort of patients with MM–associated renal impairment, the addition of TPE to contemporary anti-myeloma therapy would not result in a significant improvement in renal recovery compared with anti-myeloma therapy alone. At our center, the decision to initiate TPE is made through a multidisciplinary collaboration between hematology and nephrology teams and is primarily guided by clinical severity rather than standardized protocol criteria. TPE is generally considered in patients with severe or rapidly progressive renal dysfunction, high circulating free light-chain burden, suspected or biopsy-confirmed cast nephropathy, or urgent need for renal support. This approach reflects real-world clinical judgment and resource availability rather than an evidence-based consensus, underscoring the persistent uncertainty regarding optimal patient selection for TPE.

The present retrospective study aims to compare renal outcomes, dialysis dependence, and mortality in patients with MM-associated renal impairment treated with anti-myeloma therapy alone versus those receiving adjunctive TPE. By analyzing a decade-long experience from a tertiary care center, this study seeks to provide further insight into the clinical utility of TPE in cast nephropathy.

## 2. Materials and Methods

### 2.1. Study Design and Ethical Approval

This retrospective cohort study was conducted at Akdeniz University Hospital, a tertiary referral center in Antalya, Türkiye. The study received ethical approval from the Akdeniz University Clinical Research Ethics Committee (Approval No. 2023/164, 22 February 2023), and all procedures were performed in accordance with the Declaration of Helsinki. Medical records of patients diagnosed with MM and renal impairment between 1 January 2014 and 30 June 2023 were retrospectively reviewed.

### 2.2. Patient Selection and Eligibility Criteria

Patients were eligible for inclusion if they were aged 18 years or older, had a diagnosis of MM according to International Myeloma Working Group (IMWG) criteria, and presented with either AKI or acute-on-chronic renal impairment. Renal dysfunction at presentation was defined as serum creatinine level > 2 mg/dL or an estimated glomerular filtration rate (eGFR) < 40 mL/min/1.73 m^2^, calculated using the CKD-EPI 2021 creatinine-based equation. To ensure a clinical presentation compatible with light-chain-mediated renal injury, patients were required to have non-albuminuric proteinuria of at least 300 mg/24 h. Exclusion criteria included age under 18 years, preserved renal function (creatinine ≤ 2 mg/dL or eGFR ≥ 40 mL/min/1.73 m^2^), absence of proteinuria or presence of predominantly albuminuric proteinuria, and incomplete clinical or laboratory records.

### 2.3. Treatment Groups and Clinical Management

Patients were categorized into two groups according to the treatment they received. The first group consisted of patients who underwent TPE in addition to anti-myeloma therapy, while the second group received anti-myeloma therapy alone. The decision to initiate TPE was jointly made by nephrology and hematology teams, guided by disease severity, free light-chain burden, and renal deterioration.

### 2.4. Data Collection and Variables

**Figure 1:**
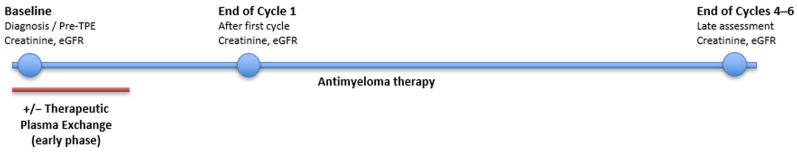
Study timeline illustrating predefined clinical assessment time points. Renal function parameters (serum creatinine and eGFR) were assessed at diagnosis (pre-TPE), at the end of the first chemotherapy cycle, and at the end of cycles 4–6. Anti-myeloma therapy was initiated promptly at diagnosis in all patients, with therapeutic plasma exchange applied selectively during the early phase in appropriate cases.

Demographic characteristics, comorbidities, baseline hematologic and biochemical parameters, serum and urine immunofixation results, involved and uninvolved free light-chain levels, and β2-microglobulin and lactate dehydrogenase (LDH) values were collected from hospital electronic records and patient charts. Patient data were collected within a predefined early treatment window encompassing the first 4–6 cycles of anti-myeloma therapy, corresponding to approximately the first 6 months after diagnosis. Renal outcomes and early mortality were assessed within this standardized observation period. Renal function parameters were evaluated at predefined clinical time points (Figure 1). Long-term follow-up beyond this window was not available for systematic analysis.

### 2.5. Therapeutic Plasma Exchange Procedure

TPE procedures were performed using continuous-flow centrifugation systems through central venous access. Anticoagulation was achieved with acid–citrate–dextrose solution A. Replacement fluid consisted primarily of 5% albumin, with isotonic saline supplementation as needed, in accordance with current American Society for Apheresis guidelines.

The number of TPE sessions, treatment intervals, and timing of discontinuation were not standardized but were individualized according to clinical response, renal function trajectory, and the treating physician’s judgment. Exchange volumes were tailored to each patient according to the calculated plasma volume. Patients were closely monitored during procedures for hemodynamic stability and electrolyte disturbances.

No major procedure-related adverse events were documented during TPE sessions. Specifically, no episodes of clinically significant hypotension or symptomatic citrate-related complications requiring medical intervention were recorded. Pre- and post-procedure laboratory parameters were systematically collected to assess the effects of TPE.

### 2.6. Kidney Biopsy Policy and Treatment Timing

Kidney biopsy was not routinely performed in all patients because of clinical instability, bleeding risk, or urgent need for treatment initiation. When feasible, biopsy was performed in patients with diagnostic uncertainty or suspected alternative causes of renal injury (*n* = 8). Consequently, a substantial proportion of patients were treated based on a clinicopathological diagnosis of light-chain-mediated renal injury rather than histologic confirmation, reflecting real-world clinical practice.

The timing of TPE and anti-myeloma therapy was determined by clinical urgency. Due to existing renal failure in all cases, anti-myeloma therapy was initiated promptly at diagnosis, with TPE started concurrently or within the early phase of treatment.

### 2.7. Anti-Myeloma Therapy

Anti-myeloma therapy regimens—typically consisting of bortezomib-based combinations with dexamethasone—were documented for all patients.

### 2.8. Study Endpoints

The primary endpoint of the study was renal response, defined as a ≥50% reduction in serum creatinine from baseline and/or the achievement of dialysis independence in patients who were receiving hemodialysis at presentation. This definition is consistent with criteria used in previous clinical investigations evaluating TPE in cast nephropathy [10,11]. Secondary endpoints included early all-cause mortality, longitudinal changes in renal function parameters, and persistence or resolution of dialysis dependence.

### 2.9. Statistical Analysis

All statistical analyses were performed using IBM SPSS Statistics version 25 (IBM Corp., Armonk, NY, USA). Continuous variables were summarized as mean ± standard deviation or median (interquartile range), as appropriate, and categorical variables as counts and percentages. Distribution of continuous variables was assessed visually and using the Kolmogorov–Smirnov test.

Baseline characteristics were compared using the independent samples *t*-test or Mann–Whitney U test for continuous variables and the chi-square or Fisher’s exact test for categorical variables, as appropriate. Between-group comparisons at predefined clinical time points were performed using the Mann–Whitney U test, while within-group changes were assessed using the Wilcoxon signed-rank test.

To account for baseline imbalances, multivariable logistic regression analysis was performed using clinically relevant variables selected a priori to minimize overfitting. Predefined subgroup and sensitivity analyses were considered exploratory. All statistical tests were two-sided, and a *p*-value < 0.05 was considered statistically significant.

### 2.10. Use of Generative Artificial Intelligence

Generative artificial intelligence (ChatGPT, OpenAI, San Francisco, CA, USA) was used solely to assist with language editing and to improve manuscript clarity. All scientific content, study design, data analysis, interpretation of results, and conclusions were independently generated, reviewed, and verified by the authors.

## 3. Results

### 3.1. Patient Characteristics

A total of 71 patients with MM and renal impairment were included in the study, of whom 41 received anti-myeloma therapy alone and 30 received TPE in addition to anti-myeloma treatment. Baseline demographic characteristics were largely similar between groups (Table 1). Mean age did not differ between the antimyeloma-only group (64.3 ± 11.0 years) and the TPE group (63.1 ± 11.4 years), and the distribution of sex and major comorbidities—including diabetes mellitus, hypertension, coronary artery disease, and hyperlipidemia—was comparable.

Paraprotein profiles were also similar, with IgG kappa representing the most frequent subtype in both cohorts. Baseline involved free light chain levels did not differ significantly between the TPE and non-TPE groups (*p* = 0.378), indicating a comparable monoclonal light-chain burden at presentation. In contrast, bortezomib-based regimens were used less frequently in patients receiving therapeutic plasma exchange than in those treated with anti-myeloma therapy alone (66.7% vs. 92.7%, *p* < 0.05). Given the pivotal role of bortezomib in rapid free light-chain reduction, this imbalance represents a potential confounder for comparative renal outcomes.

### 3.2. Baseline Renal Function

Baseline renal function differed between treatment groups. Patients receiving TPE presented with more severe renal impairment, demonstrated by a higher median serum creatinine level at diagnosis (4.28 mg/dL, IQR 3.11–5.48) compared with those treated with anti-myeloma therapy alone (2.73 mg/dL, IQR 2.25–5.10; *p* = 0.040). eGFR was numerically lower in the TPE group, although this difference did not reach statistical significance.

Hemodialysis at presentation was more frequent in the antimyeloma-only group (19.5%) than in the TPE group (3.3%); however, this difference was not statistically significant. Regardless of treatment allocation, patients requiring hemodialysis at diagnosis exhibited significantly more severe renal impairment, with higher baseline serum creatinine and lower eGFR values compared with dialysis-independent patients (creatinine: *p* < 0.001; eGFR: *p* = 0.001, Mann–Whitney U tests).

### 3.3. eGFR Trajectories

Although individual eGFR trajectories were heterogeneous, both treatment groups demonstrated a consistent improvement in median eGFR from baseline to the end of the first chemotherapy cycle, with further improvement observed by the end of cycles 4–6. Owing to variability in follow-up timing and incomplete longitudinal measurements in some patients, renal function trajectories were summarized using standardized clinical time points rather than individual patient-level plots.

Longitudinal changes in renal function and involved free light chain levels are summarized in Table 2. Within-group analyses demonstrated significant improvements in serum creatinine, eGFR, and involved free light chain levels from baseline to the end of the first cycle and to the end of cycles 4–6 in both treatment groups (Wilcoxon signed-rank test). Despite worse baseline renal function in the therapeutic plasma exchange group, the magnitude of improvement over time was similar between groups, and no significant between-group differences were observed at follow-up time points.

### 3.4. Renal Response and Dialysis Outcomes

At the end of the first chemotherapy cycle, renal response rates were similar between patients who received therapeutic plasma exchange and those who did not (40.0% vs. 36.6%, respectively; *p* = 0.964). Likewise, at the end of cycles 4–6, renal response rates did not differ significantly between patients treated with therapeutic plasma exchange and those receiving anti-myeloma therapy alone (60.0% vs. 48.7%, *p* = 0.491).

Among patients receiving TPE with available longitudinal free light chain measurements (*n* = 56), those who achieved an early renal response exhibited a greater median percentage reduction in involved free light chains compared with non-responders. Although this difference did not reach statistical significance, a borderline association was observed (Mann–Whitney U test, *p* = 0.063).

Dialysis dependence at the end of the treatment period remained comparable across groups (Table 3). In the antimyeloma-only cohort, the proportion of patients requiring dialysis declined from 19.5% at diagnosis to 14.4% after treatment. In the TPE group, dialysis requirement remained unchanged at 3.3%. The small number of dialysis-dependent patients in either group limited further comparative analysis, but there was no evidence that TPE improved dialysis independence.

### 3.5. Adjusted and Sensitivity Analyses

#### 3.5.1. Multivariable Analysis Adjusting for Baseline Imbalances

To address baseline imbalances between treatment groups, a multivariable logistic regression model was constructed including TPE, baseline serum creatinine, baseline involved free light chain levels, and use of bortezomib-based therapy. In this adjusted analysis, TPE was not independently associated with early renal response (OR 0.66, 95% CI 0.20–2.13, *p* = 0.485; Supplementary Table S1). None of the covariates included in the model were independently associated with early renal response at a statistically significant level.

#### 3.5.2. Sensitivity Analysis Restricted to Bortezomib-Based Therapy

Given the substantial imbalance in the use of bortezomib-based regimens between treatment groups, a sensitivity analysis was performed including only patients who received bortezomib-containing therapy. Within this restricted cohort (*n* = 58), early renal response was observed in 50.0% of patients treated with TPE and in 39.5% of those who did not receive plasma exchange, with no statistically significant difference between groups (χ^2^ test, *p* = 0.442).

In this same bortezomib-treated cohort, a multivariable logistic regression analysis adjusting for baseline serum creatinine and baseline involved free light chain levels (Supplementary Table S2) demonstrated that TPE was not independently associated with early renal response (OR 0.55, 95% CI 0.16–1.87; *p* = 0.337).

#### 3.5.3. Sensitivity Analysis Excluding Baseline Dialysis Dependence

To mitigate potential bias related to baseline dialysis dependence, a sensitivity analysis was conducted restricted to patients who were dialysis-independent at study entry (*n* = 62). Sustained renal response—defined as a ≥50% reduction in serum creatinine maintained at subsequent assessment—was achieved in 42.4% of patients treated with anti-myeloma therapy alone and in 41.4% of those receiving additional TPE. No significant difference in sustained renal response was observed between groups (χ^2^ = 0.007, *p* = 0.934; Fisher’s exact test *p* = 1.000).

In this dialysis-independent cohort, a subsequent multivariable logistic regression analysis adjusting for baseline serum creatinine and bortezomib-based therapy demonstrated that TPE was not independently associated with sustained renal response (OR 0.98, 95% CI 0.30–3.26; *p* = 0.975).

### 3.6. Early Mortality

During the treatment period, mortality occurred in six patients (20.0%) in the TPE group and in three patients (7.3%) in the antimyeloma-only group. Although numerically higher in the TPE cohort, this difference did not reach statistical significance (*p* = 0.239).

### 3.7. TPE Characteristics and Session Number

When free light chain reduction was analyzed, no statistically significant difference in the percentage reduction in involved FLCs was observed between patients who received TPE and those who did not (Mann–Whitney U test, *p* = 0.535). Among patients who underwent TPE, the number of TPE sessions and corresponding serum creatinine values are summarized in Table 4.

### 3.8. Association Between TPE Sessions and Renal Outcomes

The number of TPE sessions did not correlate with improvement in serum creatinine or eGFR by the end of the first chemotherapy cycle (Table 5). No significant association was observed between session number and early renal recovery.

## 4. Discussion

In this non-randomized, real-world cohort, the addition of TPE to contemporary antimyeloma therapy was not associated with an unadjusted benefit in renal recovery or dialysis outcomes. Notably, patients receiving TPE presented with more severe renal impairment at baseline, introducing a substantial risk of confounding by indication. However, even after adjustment for baseline renal function, baseline involved free light chain levels, and antimyeloma treatment in multivariable analyses, TPE remained independently unassociated with renal response.

Collectively, these findings support that renal recovery in myeloma cast nephropathy is predominantly driven by rapid and effective control of the underlying plasma cell clone rather than routine adjunctive extracorporeal light-chain removal. In our cohort, the lack of an independent renal benefit from therapeutic plasma exchange persisted even among patients receiving bortezomib-based regimens, underscoring the robustness of this observation despite baseline treatment imbalances. This aligns with contemporary real-world data demonstrating high rates of early renal recovery and low early mortality with modern bortezomib- and anti-CD38–based therapies, even in the absence of plasma exchange [12]. Expert reviews and treatment algorithms similarly emphasize that early and profound free light-chain reduction is the key determinant of renal recovery, while the role of extracorporeal light-chain removal remains controversial and is likely limited to carefully selected patients with severe or rapidly progressive disease [13,14,15,16,17].

These findings should not be interpreted as evidence against the biological plausibility of TPE across all clinical scenarios. Selected patient subgroups—such as those with biopsy-proven cast nephropathy accompanied by a very high light chain burden or those presenting early with rapidly progressive renal dysfunction—may still derive benefit from early, targeted extracorporeal strategies. Nevertheless, such hypotheses require confirmation in prospective, biopsy-guided, and risk-stratified clinical trials.

Overall, our results suggest that in routine real-world practice, the indiscriminate use of TPE does not confer additional clinical benefit beyond that achieved with contemporary antimyeloma therapy.

Our results are consistent with the landmark randomized controlled trial by Clark et al. [9], which similarly demonstrated no reduction in mortality, dialysis dependence, or persistent renal impairment among patients receiving TPE in addition to conventional therapy. In contrast, earlier single-center studies and observational cohorts have reported more favorable outcomes, suggesting improvements in renal function and survival with TPE use. For example, Leung et al. [10] reported an association between TPE and improved renal recovery, particularly among patients with biopsy-proven cast nephropathy and substantial free light chain reduction. Similarly, Premuzic et al. [6] observed clinical benefit when TPE was initiated early in the course of kidney injury. These divergent findings may be attributable to heterogeneity in patient selection, baseline renal severity, timing of TPE initiation, and rates of biopsy confirmation.

Mortality did not differ significantly between treatment groups, although it was numerically higher in the TPE cohort, likely reflecting the more severe clinical presentation of patients selected for TPE. Alternatively, the absence of a mortality benefit may indicate that TPE does not substantially modify the natural course of myeloma-associated acute kidney injury, particularly in the era of contemporary antimyeloma therapy. The lack of an observed association between the number of TPE sessions and renal or early survival outcomes further supports the limited incremental value of intensifying extracorporeal treatment.

This study has several limitations that should be acknowledged. First, its retrospective and non-randomized design introduces inherent selection bias, as patients receiving TPE tended to present with more severe renal impairment at baseline. Although this imbalance was addressed through adjusted multivariable analyses, residual confounding cannot be fully excluded. Second, the diagnosis of myeloma-related cast nephropathy was primarily based on clinical and laboratory criteria, as kidney biopsy was not routinely performed, potentially leading to diagnostic heterogeneity. Third, although longitudinal improvements in renal function and involved free light chain levels were formally demonstrated, follow-up was limited to the initial 4–6 cycles of therapy, precluding comprehensive assessment of long-term renal and survival outcomes. Finally, the single-center design and relatively small sample size may limit the generalizability of the findings. Despite these limitations, this study reflects real-world clinical practice and provides clinically relevant insights into the role of TPE in the era of contemporary antimyeloma therapy.

## 5. Conclusions

In this real-world cohort, adjunctive TPE was not independently associated with improved early renal recovery or dialysis independence beyond that achieved with contemporary antimyeloma therapy. These findings underscore that effective antimyeloma treatment remains the primary driver of renal recovery. Although TPE may have a role in carefully selected patients, the routine use of TPE in unselected cases is not supported by the present data.

## Figures and Tables

**Table 1:** Baseline Demographic and clinical characteristics of the patients.

Variable	Anti-Myeloma Treatment Only (*n* = 41)	TPE + Anti-Myeloma Treatment (*n* = 30)
Continuous Variables (mean ± SD)		
Age (years)	64.32 ± 11.0	63.07 ± 11.36
Hemoglobin (g/dL)	8.84 ± 1.74	9.39 ± 1.62
Leukocyte count (/mm^3^)	7707.8 ± 5142.53	8411.43 ± 11,950.59
Neutrophil count (/mm^3^)	4647.8 ± 2735.87	4092.33 ± 2030.56
Platelet count (×10^3^/mm^3^)	179.66 ± 61.96	185.8 ± 96.05
24 h urine protein (mg/24 h)	2673.78 ± 2669.17	3257.97 ± 2612.00
24 h urine albumin (mg/24 h)	331.82 ± 1005.47	121.54 ± 174.93
Calcium (mg/dL)	10.42 ± 2.17	10.09 ± 1.98
β2-microglobulin (mg/L)	19.16 ± 20.54 (*n* = 40)	14.75 ± 12.09
LDH (U/L)	255.39 ± 121.96	277.3 ± 219.33
Categorical Variables, *n* (%)		
Hemoglobin levels		
<10 g/dL	29 (70.7%)	18 (60.0%)
≥10 g/dL	12 (29.3%)	12 (40.0%)
Calcium levels		
<11 mg/dL	28 (68.3%)	23 (76.7%)
≥11 mg/dL	13 (31.7%)	7 (23.3%)
β2-microglobulin groups		
<3.5 mg/L	3 (7.5%)	2 (6.7%)
3.5–5.5 mg/L	8 (20.0%)	3 (10.0%)
≥5.5 mg/L	29 (72.5%)	25 (83.3%)
LDH levels		
Normal	19 (46.3%)	16 (53.3%)
High	22 (53.7%)	14 (46.7%)
ISS stage		
Stage I	2 (5.0%)	2 (6.7%)
Stage II	8 (20.0%)	3 (10.0%)
Stage III	30 (75.0%)	25 (83.3%)
Bortezomib-based regimen	38 (92.7%)	20 (66.7%)

Continuous variables are presented as mean ± standard deviation; categorical variables are shown as number (percentage). Abbreviations: TPE, therapeutic plasma exchange; LDH, lactate dehydrogenase; ISS, International Staging System.

**Table 2:** Renal function and free light chain dynamics according to treatment group.

Variable	Anti-Myeloma Treatment Only (*n*_1_ = 41)	TPE + Anti-Myeloma Treatment (*n*_2_ = 30)	Between Group *p*-Value
Serum creatinine (mg/dL), median (IQR)			
Baseline (Diagnosis/Pre-TPE)	2.73 (2.25–5.10)	4.28 (3.11–5.48)	**0.040**
End of 1st cycle	1.80 (1.07–3.10)	1.88 (1.19–3.70)	0.834
End of 4–6th cycles	1.37 (0.80–2.33)	1.35 (0.80–3.02)	0.841
Within-group *p*-value	**<0.001**	**<0.001**	—
eGFR (mL/min/1.73 m^2^), median (IQR)			
Baseline (Diagnosis/Pre-TPE)	22 (10–29)	13 (9–21)	0.053
End of 1st cycle	32 (16–62)	31.5 (18–64)	0.963
End of 4–6th cycles	49 (28–89)	46 (22–91)	0.685
Within-group *p*-value	**<0.001**	**<0.001**	—
Involved free light chain (mg/L), median (IQR)			
Baseline (n_1_/n_2_ = 36/27)	132 (15–830)	121 (15–998)	0.378
End of 1st cycle	20.1 (5.6–114.3)	24.6 (5.4–116.7)	0.476
End of 4–6th cycles	24.6 (3.2–64.5)	14.7 (3.2–177.9)	0.714
Within-group *p*-value	**0.001/0.005** *	**0.006/0.037** *	—

Between-group comparisons were performed using the Mann–Whitney U test. Within-group changes from baseline were assessed using the Wilcoxon signed-rank test. For involved free light chain levels, within-group *p*-values are reported for baseline → end of 1st cycle and baseline → end of cycles 4–6, respectively. eGFR was calculated using the CKD-EPI equation. Abbreviations: TPE, therapeutic plasma exchange; eGFR, estimated glomerular filtration rate. * Within-group *p*-values correspond to baseline → end of 1st cycle and baseline → end of cycles 4–6, respectively.

**Table 3:** Hemodialysis requirement at diagnosis and end of treatment.

Variable	Anti-Myeloma Treatment Only (*n* = 41)	TPE + Anti-Myeloma Treatment (*n* = 30)	*p*-Value
Hemodialysis at diagnosis, *n* (%)	8 (19.5%)	1 (3.3%)	0.069
Hemodialysis at end of treatment, *n* (%)	6 (14.6%)	1 (3.3%)	0.226

Values shown as number (percentage). *p*-values obtained by Fisher’s exact test.

**Table 4:** Changes in serum creatinine during sequential TPE sessions.

TPE Session	*n*	Median (IQR)	Min–Max
Pre-procedure creatinine	30	4.16 (3.11–5.16)	1.72–7.74
After 1st session	30	3.38 (2.62–4.20)	1.57–6.64
After 2nd session	27	3.15 (2.17–4.02)	1.40–6.53
After 3rd session	18	2.88 (1.87–3.40)	1.58–6.04
After 4th–6th sessions	16	2.18 (1.30–3.30)	0.80–6.00
After 10th session	5	1.43 (1.43–3.21)	0.90–4.82
After 12th–14th sessions	2	3.93 (2.94–4.91)	2.94–4.91

Descriptive creatinine values across sequential sessions (available cases).

**Table 5:** Association between number of TPE sessions and renal function response.

Variable	*n*	Number of TPE Sessions, Median (IQR)	*p*-Value
**Creatinine response**			0.824
No decrease	18	4 (2–6)	
Decrease	12	3 (2–7)	
**eGFR response**			0.518
No increase	9	3.5 (2–6)	
Increase	21	5 (2–7.5)	

Association tested using Mann–Whitney U test. No significant correlation observed.

## Data Availability

The data supporting the findings of this study are available from the corresponding author upon reasonable request.

## Supplementary Materials

The following supporting information can be downloaded at: https://www.mdpi.com/article/10.3390/jcm15020417/s1, Table S1. Adjusted Multivariable Logistic Regression Analysis for Early Renal Response at the End of the First Chemotherapy Cycle; Table S2. Multivariable logistic regression analysis for early renal response among patients receiving bortezomib-based therapy.
