# Supplementary material for: The Role of Therapeutic Plasma Exchange in the Management of Myeloma-Related Cast Nephropathy: A 10-Year Real-World Cohort Study
Source: J Clin Med. 2026 Jan 6;15(2):417. doi: 10.3390/jcm15020417 (PMC12842403; doi:10.3390/jcm15020417)
Supplement: Supplementary file 1 [file jcm-15-00417-s001.zip › jcm-4057247-supplementary.pdf]

Supplementary Table S1. Adjusted Multivariable Logistic Regression Analysis for Early Renal Response at the End of the First Chemotherapy Cycle

Variable	Odds Ratio (OR)	95% CI (Lower–Upper)	p-value
**Therapeutic plasma exchange (yes vs. no)**	0.66	0.20 – 2.13	0.485
**Baseline involved free light chain level (mg/L)**	1.00	1.00 – 1.00	0.860
**Baseline serum creatinine (mg/dL)**	0.83	0.62 – 1.12	0.219
**Bortezomib-based therapy (yes vs. no)**	0.27	0.05 – 1.48	0.131

Outcome: Early renal response at the end of cycle 1 (yes vs. no), Model: Binary logistic regression (Enter method), Sample size: n = 63, Model fit: Hosmer–Lemeshow p = 0.169; Nagelkerke R^2 = 0.086, Odds ratios >1 indicate a higher likelihood of achieving early renal response. Baseline involved free light chain levels were entered as continuous variables. The model was constructed to account for baseline renal imbalance and treatment heterogeneity.

Supplementary Table S2. Multivariable logistic regression analysis for early renal response among patients receiving bortezomib-based therapy

Variable	OR	95% CI	p-value
**Therapeutic plasma exchange**	0.55	0.16–1.87	0.337
**Baseline serum creatinine (mg/dL)**	0.84	0.63–1.14	0.269
**Baseline involved FLC (mg/L)**	1.00	1.00–1.00	0.654
